# Bacteriotherapy for inflammatory bowel disease

**DOI:** 10.1186/s41232-020-00153-4

**Published:** 2021-01-13

**Authors:** Yusuke Yoshimatsu, Yohei Mikami, Takanori Kanai

**Affiliations:** Division of Gastroenterology and Hepatology, Department of Internal Medicine, Keio University School of Medicine, 35 Shinanomachi, Shinjuku-ku, Tokyo, 160-8582 Japan

**Keywords:** Dysbiosis, *Clostridioides* (*Clostridium*) *difficile* infection, Fecal microbiome transplantation, Prebiotics, Probiotics, Bacteriotherapy

## Abstract

The number of patients with inflammatory bowel disease is rapidly increasing in developed countries. The main cause of this increase is thought not to be genetic, but secondary to rapidly modernized environmental change. Changes in the environment have been detrimental to enteric probiotics useful for fermentation, inducing an increase in pathobionts that survive by means other than fermentation. This dysregulated microbiota composition, the so-called dysbiosis, is believed to have increased the incidence of inflammatory bowel disease. Bacteriotherapy, a treatment that prophylactically and therapeutically corrects the composition of disturbed intestinal microbiota, is a promising recent development. In fact, fecal microbiome transplantation for recurrent *Clostridioides difficile* infection in 2013 was a significant contribution for bacteriotherapy. In this paper, we comprehensively review bacteriotherapy in an easy-to-understand format.

## Highlights

- Dysbiosis is considered as one of the major causes of intestinal diseases and beyond.
- FMT is an alternative treatment option for patients with rCDI.
- Bacteriotherapies are studied for the treatment of intestinal dysbiosis.

## Introduction

Humans have consistently consumed fermented food for a long time. Fermenters, types of bacteria known as “probiotics,” allowed the invention of fermented food. It is thought that fermented foods were initially developed as a means of protecting food from corruption, but not for the purpose of health maintenance, disease prevention, or treatment. In 1911, the Russian immunologist Metchnikoff advocated probiotics’ agelessness theory that health could be enhanced and senility delayed by manipulating the intestinal microbiome with host-friendly bacteria found in yogurt because Bulgarians who consumed large amounts of yogurt had long life spans [1]. Fermentation was a prototypical example of the symbiotic relationship between humans and the gut microbiome, further forming a beneficial intestinal environment, including barrier function and immunity [2–7]. However, these symbiotic ties have gradually grown weaker since the invention of the antibiotic penicillin. Additionally, humans invented what was considered a “magical box” at the time, the refrigerator. The refrigerator enabled longer food preservation without any complex preparation and subsequently reduced the consumption of fermented food. Decreased fermented food intake was linked to decreased intake of dietary fibers, while concurrently, the intake of animal fat and protein gained popularity in developed countries.

Over the last several decades, there has been a health boom in developed countries, and consumption of fermented foods, such as yogurt, has again grown in popularity. However, there is insufficient evidence to prove the beneficial effects of “health foods” including prebiotic or probiotic foods. Prebiotics are a rich source of fibers and natural sugars and harbor beneficial bacteria in the gut; as such, most of them are categorized as health foods, but not medicines [8, 9]. However, recurrent *Clostridioides* (formerly *Clostridium*) *difficile* infection (rCDI) which is resistant to antibiotics emerged as a serious threat to public health in the late twentieth century in Europe and the USA. Given the therapeutic success of fecal microbiome transplantation (FMT) in treating rCDI, bacteriotherapy is a hot topic among researchers and clinicians worldwide [10].

### Microbiota

**Fig. 1:**
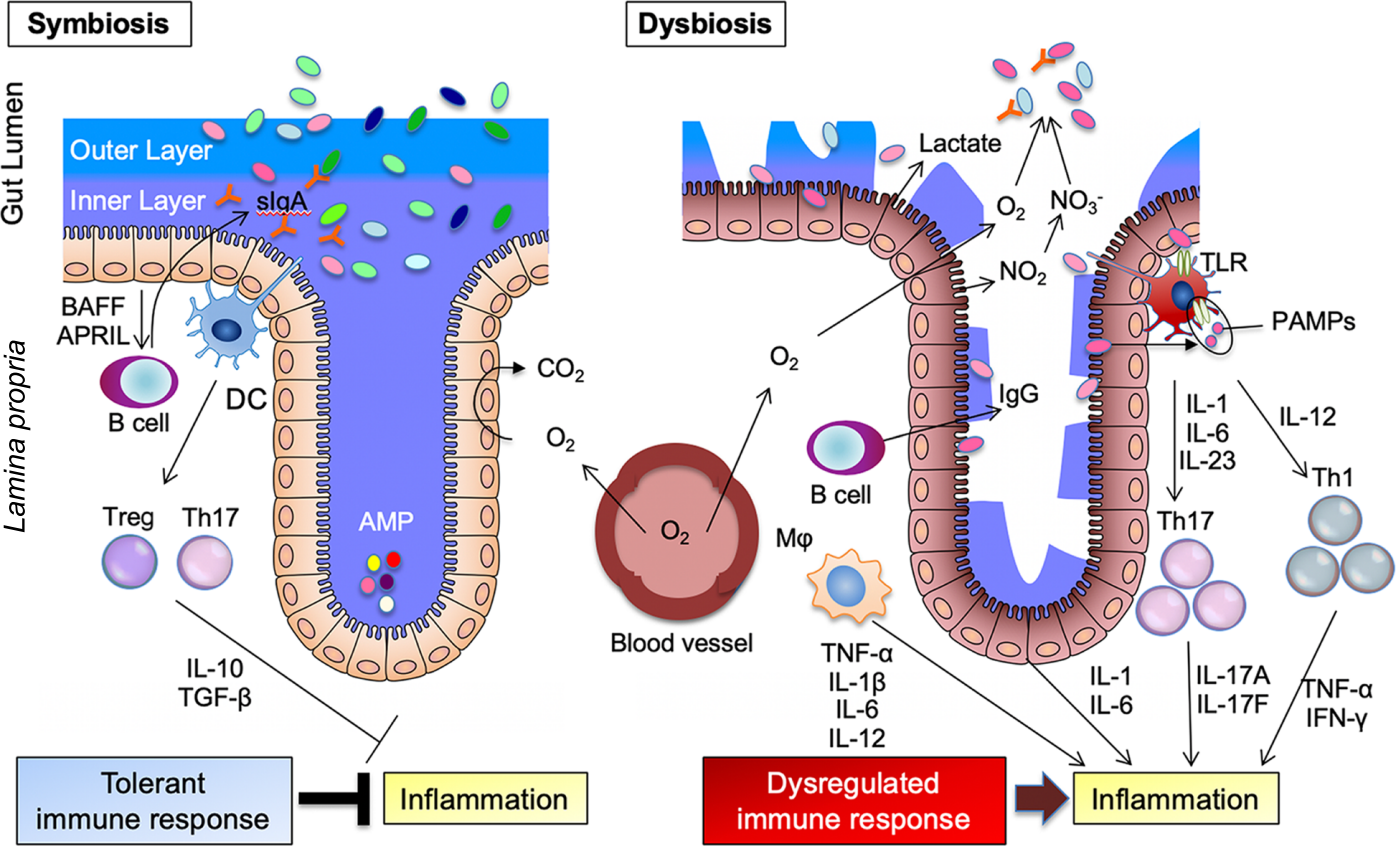
Gut microbiota regulates immune response. Symbiosis (left): The intestinal immune system within the gut lamina propria becomes largely tolerant to the resident commensals under tight control by intestinal epithelial cells with steady-state proportions of mucus, secretory IgA, and antimicrobial peptides. As a result, the microbiota stays in the intestinal lumen. The gut microbiome stimulates intestinal epithelial cells, T cells, and lamina propria dendritic cells (DCs) and macrophages to promote the development or the activation of forkhead box P3(Foxp3) positive Treg cells. The others activate lamina propria DCs and macrophages to induce T helper 17 (Th17) cells. Toll-like receptor (TLR) activation on intestinal epithelial cells induces the secretion of B cell-activating factor (BAFF) and A proliferation-inducing ligand (APRIL), which promote the differentiation of IgA-producing plasma cells. CD103-positive DCs support the development of Tregs to secrete IL10 and TGF-β, and they also stimulate B cells to produce commensal-specific IgA. Dysbiosis (right): Dysbiosis is a condition characterized by a loss of symbiotic bacteria and overgrowth of pathobionts. Pathobiont overgrowth leads to the loss of barrier integrity and a breach in the intestinal epithelial cell barrier. Translocation of pathobionts, bacterial components, and diverse pathogen-associated molecular patterns (PAMPs) triggers the intestinal immune system through TLR activation, resulting in chronic inflammation involving hyperactivation of T helper 1 (Th1) and Th17 cells. The secretion of inflammatory cytokines from intestinal epithelial cells exacerbates a Th1 and Th17 response by DCs and macrophages and leads to the accumulation of commensal-specific IgG by B cells. Additionally, disruption of the gut microbiota changes the metabolism of colonocytes, which is characterized by low oxygen consumption and generation of nitric oxide (NO) followed by synthesis of inducible nitric oxide synthase (iNOS). NO converts into nitrate (NO_3_^−^) in the intestinal lumen and drives the expansion of facultative anaerobic bacteria, including pathobionts with excessive oxygen (O_2_)

Humans are in symbiosis with the microbiota in the GI tract, with over 100 trillion microbiota, including 1000 types and 1 million associated genes [11]. Technological innovations in genetic analysis have shown that failure of the human-enteric symbiosis ecosystem, the so-called dysbiosis, is closely related to growing disease groups, including inflammatory bowel disease (IBD) in developed countries (Fig. 1) [12–14]. Although microbiota can be as simple as parasites that cling to nutrition from human host or “friendly” bacteria that colonize the gut to assist fermentation, the Westernized lifestyle has made harboring these “good” bacteria in the intestine more difficult. This lifestyle refers to inappropriate use of antibiotics [15], cesarean section [16], artificial milk [17], improved hygiene [18], high-fat and low-fiber diet [19], and stress [20], which all can cause dysbiosis. In particular, dietary fiber is an important nutrient for these probiotic bacteria [21], and the lack of dietary fiber intake in modern society is considered the primary cause of dysbiosis in the gut.

### Microbiota in IBD

Many previous studies have shown environmental factors, such as modern lifestyle, diet, and antibiotic use, are associated with the development of IBD [5, 22–25]. Particularly, microbiota plays critical roles in pathogenesis of IBD together with genetic background and immunological factors. Utilizing the next-generation sequencing (NGS) technology, recent studies have profiled gut microbiota at high resolution and indicated reduced diversity of the gut microbiota in IBD patients [26–30]. For example, Frank et al. [31] demonstrated that the abundances of bacterial members of the phyla *Firmicutes* and *Bacteroidetes* are reduced and those of Actinobacteria and Proteobacteria are increased in IBD. In addition, Sokol et al. [32, 33] demonstrated that the phylum *Firmicutes*, and particularly a member of *Clostridium* cluster IV, *Faecalibacterium prausnitzii*, is reduced in the feces of Crohn’s disease patients. Consistently, later landmark studies have revealed 17 strains within clusters IV, XIVa, and XVIII of *Clostridia* induces regulatory T cells (Tregs) in the gut [34, 35]. Contrary to the immunosuppressive roles of specific bacteria, no specific bacteria has not been proved to do harm with IBD patients. For example, *Mycobacterium avium* ssp. *paratuberculosis* has long been suspected to have a pathogenic effect on Crohn’s disease in a manner analogous to granuloma-forming mycobacterium infectious diseases [36]. However, an antitubercular drug administered to Crohn’s disease patients revealed no efficacy in a 2-year clinical trial [37]. Then, the relationship between other bacterial genera, such as *Listeria* and *Mycoplasma*, and Crohn’s disease has been investigated [38, 39]. However, recent studies have revealed possible interaction of “pathogenic” bacteria and breaking down intestinal homeostasis. Adherent-invasive *Escherichia coli* (AIEC) has been isolated from ileal biopsy samples from patients with Crohn’s disease. AIEC can be pathogenic by adhering and invading intestinal epithelial cells [40, 41]. AIEC also can replicate in macrophages and stimulate production of tumor necrosis factor (TNF)-a by macrophages [41]. *Fusobacterium varium* has been known to attach to inflamed mucosa and invade ulcerated mucosa in ulcerative colitis patients [42]. Besides focusing on these specific microorganisms, alteration in the composition of gut microbiota has been also highlighted regarding with the pathogenesis of IBD or predication of disease course. *F. prausnitzii* is associated with the increased risk of postoperative recurrence in CD patients [33]. We recently demonstrated that 5-ASA intolerance is associated with a risk of adverse clinical outcomes and dysbiosis [43]. It is of note that computational analyses of gut microbiome allow to predict the efficacy of a certain biologic agent on UC [44].

In summary, the gut homeostasis is maintained by the balanced diverse microbiota and the possible “good” bacteria including members of *clostridia*, while some specific bacteria might be associated with developing IBD. However, it remains controversial whether the IBD-associated dysbiosis in the gut microbiota is a cause or a consequence of intestinal inflammation.

### Prebiotics

Dietary fibers, unlike starches, are carbohydrates with complex sugar chain structures. In recent years, the importance of dietary fiber has been noted, but humans themselves do not possess digestive enzymes that process dietary fibers. Dietary fiber can be digested in the body by probiotics that carry digestive enzymes, and individual dietary fiber molecules are collectively called prebiotics. Thus, prebiotics are important nutrient sources for probiotics growth. Probiotics not only proliferate using prebiotics as a nutrient source, but also produce beneficial metabolites, such as short-chain fatty acids (SCFA)—propionate, acetate, butylate—that play an important role in maintaining homeostasis of the host while metabolizing prebiotics [45]. In modern society, where the intake of dietary fibers is insufficient, treatment with supplementing prebiotics is a strategy to increase probiotics, and many attempts have been made for various diseases [46–50]. At present, although there is a large amount of evidence in animal models [51–55], clinical trials have been unsatisfactory. It is possible that in these studies, the doses were too small, the dosing period was too short, or the scale of the clinical trial was too small. Healthy individuals are exposed to health information now more than ever, such as the negative effects of a diet lacking in vegetables. However, some of this information, such as fashionable promotion of organic vegetables, is not fully supported by current scientific studies.

### Probiotics

**Fig. 2:**
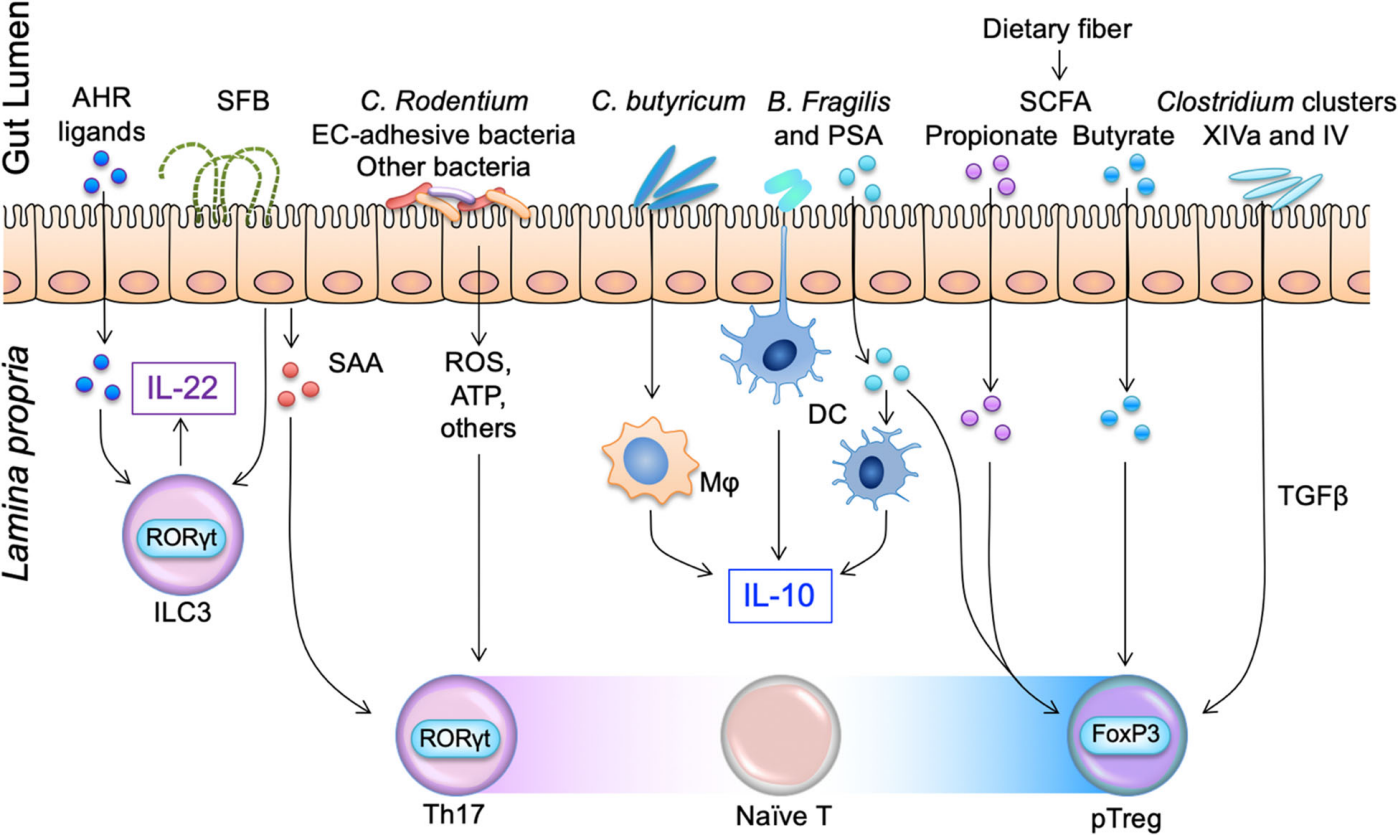
Prebiotics, probiotics, and postbiotics each have specific associations with immune cells in the lamina propria. Aryl hydrocarbon receptor (AHR) ligands, which are present in food antigens, promote mucosal healing via IL-22 production from group 3 innate lymphoid cells (ILC3). SFB also promote IL-22 production from ILC3 and activate lamina propria DCs and macrophages to induce Th17 cells through the production of SAA from IECs. *C. rodentium* and epithelial cell (EC)-adhesive bacteria induce Th17 differentiation by reactive oxygen species (ROS), adenosine triphosphate (ATP), and others. *C. butyricum* induce intestinal IL-10-producing macrophages, while polysaccharide A (PSA)-positive *B. fragilis* stimulate DCs to produce IL-10. PSA itself directly promotes the development of the activation of Foxp3-positive Treg cells in a similar way to SCFA, such as propionate and butyrate. *Clostridium* clusters IV and XIVa also promote Treg cell accumulation via TGF-β production from IEC

Probiotics produce metabolites that are crucial for gut homeostasis. Examples of probiotic foods include yogurt, kimchi, and cheeses. With the recent boom in health information, some people have increased their intake, but this intake is likely much smaller than that of 100 years ago. We speculate that the emergence of the refrigerator—the “great invention” that changed the modern lifestyle—is critically involved in the dysbiosis in Western people. Until the eighteenth century, human beings consumed a large amount of fermented food, because fermenting was one of the major ways to preserve food, similar to salting, spicing, and smoking. Therefore, the ancient human was constantly exposed to fermenting bacteria by ingesting fermented food. However, the invention of the refrigerator enabled humans to preserve food easily, and the time-consuming process of fermented food was less common than it was in the “pre-refrigeration era.” At present, as with prebiotics, there is a large amount of evidence in animal models [33, 35, 56–58] (Fig. 2) on the benefits of probiotics, but little evidence in clinical trials for human disease. Additionally, studies on intake of probiotics without prebiotics is likely counterproductive; fortunately, in recent years, both probiotic and prebiotic treatments (symbiotics) have been tried. Kimchi, a type of Chinese cabbage (prebiotics), and lactic acid bacteria (probiotics) are examples of symbiotics [59]. Though often forgotten, it is important to note that in probiotic foods (such as yogurt) sold today in health stores, isolated bacteria are present, rather than human-derived bacteria. Gordon et al. demonstrated that mouse gut bacteria, rather than human-derived bacteria, termite-derived bacteria, and soil-derived bacteria, have the best colonization efficiency for the murine intestinal tract [60]. Naturally, probiotics derived from human feces are expected to be the most compatible with human gastrointestinal tracts and have a noticeable therapeutic effect in treatment strategies related to human health and diseases. Unfortunately, human-derived feces are not easily adapted for patients because of a general perception of uncleanliness.

### FMT

FMT has recently attracted attention in the medical field. FMT is a method of transplanting the microbiota in the feces of a healthy donor into the intestines of sick patients. FMT has a surprisingly long history and was described in China in the fourth century. In Chinese medical books, treatments with administration of feces from healthy donors to cure food poisoning are described. In 1958, one surgeon named Eisman performed FMT on four patients with pseudomembranous enteritis in the USA [61]. However, with the discovery and clinical application of various antibiotics (starting with penicillin), FMT was forgotten and only sporadically mentioned in the medical community. In the 1990s, *Clostridioides difficile* 027/BI/NAP1 mutant strains, which produced high levels of toxin A, resisted antibiotics, and infections often became recurrent and fatal. Numerous cases broke out in North America and became a serious social problem. In 2013, a Dutch group reported the overwhelming efficacy of the first randomized controlled trial (RCT) for recurrent CD infection (CDI) [10].

FMT is a direct example of clinically applied bacteriotherapy and became popular with the spread of microbiota analysis using next-generation sequencing technology called 16S rRNA sequencing. Numerous RCTs have proven FMT efficacy against rCDI, and meta-analysis also demonstrated their effectiveness [62, 63]. In 2016, the Food and Drug Administration (FDA) has approved the use of FMT after rigorous informed consent and the approval of the ethical review board at the given healthcare facility. In North America, there is a drive to encapsulate feces, and promising results have been obtained treating rCDI with orally administered fecal capsules [64]. Fecal capsules promote the spread of FMT, but it is unclear whether processed capsules qualify under the Investigational New Drug (IND) application.

A German group, in contrast, took a unique therapeutic approach with five rCDI patients. First, third-party donor-derived bacteria were suspended in saline, filtered to prevent the passage of bacteria, and administered as a non-bacterial supernatant to the patient (fecal filtrate transfer; FFT). Surprisingly, in all 5 patients, FFT restored normal stool habits and eliminated symptoms of CDI, suggesting that there is no need for conventional FMT—but, the supernatant contained a critical healing factor other than bacteria in donor feces [65]. It is possible that the critical curing factor was a metabolite or bacteriophage produced by microbiota derived from healthy donors. At present, FMT is being tested for various diseases associated with dysbiosis [66–74]. As for efficacy, in particular, it remains controversial whether FMT has a beneficial effect on active UC or not. An Australian group demonstrated that intensive-dosing (5 days per week for 8 weeks), multi-donor (between three and seven unrelated donors) FMT induces clinical remission and endoscopic improvement in active UC [66]. To the contrary, our group demonstrated that single FMT using feces derived from single-donor of each patients’ relative has a limited effect on UC in Japanese patients [74]. This discrepancy may occur due to difficulty in restoring bacterial flora by single administration. On the other hand, as for safety, the establishment of FMT must reiterate the safety of donor feces, the mechanism of the symbiotic ecosystem between microbiota and human host, the metabolites produced by microbiota, and the other microorganisms, such as bacteriophages and fungi, coexisting in the feces.

### Defined microbiota

The long-term safety of donor feces for use during FMT is still unclear. Just because the donor is a healthy person, one cannot conclude that the donor feces do not have unknown infectious agents. A Canadian group is mass-cultivating anaerobic probiotics from the feces of healthy donors using “Robogut,” a robot that mimics the conditions in the colon; the group has developed a high-volume probiotics suspension composed of 33 types of bacteria [75]. It has already been reported that the bacterial cocktail shows positive effects in rCDI patients, although the trial is small [76]. These researchers used a precise process to prevent the contamination of pathogens, as with yogurt. While the safety concerns in FMT, defined microbiota, which uses probiotics from processed feces for therapeutic purposes, is treated as a drug; it therefore must be strictly studied under clinical development regulations and with an Investigational New Drug (IND) application. In 2016, the FDA revised guidelines for bacterial therapy for pharmaceutical purposes, which were different from conventional treatment with healthy food such as yogurt.

### Postbiotics

With the rapid accumulation of information on genes (metagenomics) and metabolites (metabolomics) related to microbiota, researchers are attempting to apply the metabolite produced by microbiota itself. This process is called “postbiotics,” and research has been actively promoted in recent years. Other examples of postbiotics include molecules that modify metabolite production. For example, a molecule that inhibits an enzyme that produces trimethylamine N-oxide (TMAO), which promotes arteriosclerosis, is being researched as a postbiotic [77]. While these approaches are in the early stages, pharmaceutical research is rapidly growing.

### Probiotics and gut immunity

It is known that the gut microbiome has a close relationship with immune cells in the lamina propria and substantially contributes to intestinal diseases [2, 4, 6, 12, 14, 78–80]. Each probiotic bacterial strain shapes unique characteristics of the immune response (Fig. 2). In one mouse model, segmented filamentous bacteria (SFB) IL-17A produce Th17 cells through the production of serum amyloid A (SAA) from intestinal epithelial cells (IEC) [81]. Some microbiota belonging to the genus *Clostridium* induce regulatory T cells (Treg) by producing butyrate from dietary fiber, and in turn, the butyrate suppresses inflammatory cytokines via mucin and antimicrobial peptide from IEC [4, 34, 35, 82, 83]. Consistently, another butyrate-producing *Clostridium* strain, *Clostridium butyricum* MIYAIRI 588, suppresses colitis via IL-10 production from intestinal macrophages and TGF-beta from dendritic cells [5, 56]. These findings are consistent with those of recent human studies. The fecal butyrate levels and the proportion of *Clostridia* are significantly lower in patients with ulcerative colitis (UC) than in healthy controls [31, 84, 85]. We recently reported the efficacy of herbal medicine, indigo naturalis, in patients with active UC, which is accompanied by corrected gut microbiota [29]. We speculate that therapeutic agents currently used in the clinic partially, but significantly, promote “beneficial” microbiota.

## Conclusion

In the near future, the mechanisms of symbiosis in various microorganisms that coexist in the intestinal environment, including intestinal microbiota, will be clarified. Future medical practice may use bacteriotherapy, which is independent of a fecal bank and applies more refined microbiota. Additionally, molecules produced by microbiota genes may appear as pharmaceuticals. At present, it is desirable to use bacteriotherapy for a group of life-threatening diseases, such as rCDI. However, for patients with non-fatal and intractable diseases, the bacteriotherapy could be challenged because lack of its safety evidence. A future of bacteriotherapy that is in harmony with the development of various drugs is desired.

## Data Availability

Not applicable.

## Abbreviations

AHR: Aryl hydrocarbon receptor
APRIL: A proliferation inducing ligand
ATP: Adenosine triphosphate
BAFF: B cell-activation factor
DCs: Dendritic cells
EC: Epithelial cells
FDA: Food and Drug Administration
FMT: Fecal microbiome transplantation
Foxp3: Forkhead box P3
IBD: Inflammatory bowel disease
IEC: Intestinal epithelial cells
IEC: Intestinal epithelial cells
ILC3: Group 3 innate lymphoid cells
IND: Investigational New Drug
iNOS: Inducible nitric oxide synthase
NO: Nitric oxide
NO_3_^−^: Nitrate
O_2_: Oxygen
PAMPs: Pathogen-associated molecular patterns
PSA: Polysaccharide A
rCDI: Recurrent *Clostridioides* (formerly *Clostridium*) *difficile* infection
RCT: Randomized controlled trial
ROS: Reactive oxygen species
Th1: T helper 1
Th17: T helper 17
TLR: Toll-like receptor
TMAO: Trimethylamine N-oxide
Treg: Regulatory T cells
SAA: Serum amyloid A
SCFA: Short-chain fatty acids
SFB: Segmented filamentous bacteria
UC: Ulcerative colitis
